# A novel *TCF7L2* type 2 diabetes SNP identified from fine mapping in African American women

**DOI:** 10.1371/journal.pone.0172577

**Published:** 2017-03-02

**Authors:** Stephen A. Haddad, Julie R. Palmer, Kathryn L. Lunetta, Maggie C. Y. Ng, MEDIA Consortium, Edward A. Ruiz-Narváez

**Affiliations:** 1 Slone Epidemiology Center at Boston University, Boston, MA, United States of America; 2 Department of Biostatistics, Boston University School of Public Health, Boston, MA, United States of America; 3 Center for Genomics and Personalized Medicine Research, Center for Diabetes Research, Wake Forest School of Medicine, Winston-Salem, NC, United States of America; Children's National Health System, UNITED STATES

## Abstract

SNP rs7903146 in the Wnt pathway’s *TCF7L2* gene is the variant most significantly associated with type 2 diabetes to date, with associations observed across diverse populations. We sought to determine whether variants in other Wnt pathway genes are also associated with this disease. We evaluated 69 genes involved in the Wnt pathway, including *TCF7L2*, for associations with type 2 diabetes in 2632 African American cases and 2596 controls from the Black Women’s Health Study. Tag SNPs for each gene region were genotyped on a custom Affymetrix Axiom Array, and imputation was performed to 1000 Genomes Phase 3 data. Gene-based analyses were conducted using the adaptive rank truncated product (ARTP) statistic. The *PSMD2* gene was significantly associated with type 2 diabetes after correction for multiple testing (corrected p = 0.016), based on the nine most significant single variants in the +/- 20 kb region surrounding the gene, which includes nearby genes *EIF4G1*, *ECE2*, and *EIF2B5*. Association data on four of the nine variants were available from an independent sample of 8284 African American cases and 15,543 controls; associations were in the same direction, but weak and not statistically significant. *TCF7L2* was the only other gene associated with type 2 diabetes at nominal p <0.01 in our data. One of the three variants in the best gene-based model for *TCF7L2*, rs114770437, was not correlated with the GWAS index SNP rs7903146 and may represent an independent association signal seen only in African ancestry populations. Data on this SNP were not available in the replication sample.

## Introduction

African American women experience a greater burden from type 2 diabetes compared to U.S. women of European ancestry. Incidence in African American women is more than twice that in U.S. white women, with >50% of this excess rate remaining after adjustment for known type 2 diabetes risk factors including body mass index (BMI) [1]. In addition, African Americans with diabetes have poorer glycemic control [2] and an increased risk of diabetic complications and mortality [3] compared to whites. Given these racial disparities, it is critical that more studies be conducted to investigate the etiology of type 2 diabetes in African American women.

More than 75 genetic loci for type 2 diabetes have been discovered in European, Asian, and Mexican ancestry populations [4–7], while only three novel variants have been discovered in genome-wide association studies (GWAS) of African ancestry (AA) populations [8,9]. Attempts to replicate type 2 diabetes associations from European samples in AA populations suggest that a majority of the variants show associations in the same direction in AA samples [8,10–13]. However, only a few loci have achieved statistical significance in replication attempts. Most notable is SNP rs7903146 in the *TCF7L2* gene, the variant most significantly associated with type 2 diabetes to date.

*TCF7L2* encodes a transcription factor that plays an important role in the Wnt signaling pathway, and its risk alleles appear to be associated with impaired insulin secretion / beta-cell function [14,15]. The Wnt pathway is one of the cell’s most important developmental and growth regulatory mechanisms [16], critical in determining cell fate, proliferation, polarity, and cell death during embryonic development, and also in adult tissue homeostasis. Abnormalities in Wnt signaling have been implicated in a variety of human diseases [17].

The Wnt signaling pathway is actually a group of signal transduction pathways: the canonical Wnt pathway leads to the regulation of gene transcription, and multiple non-canonical Wnt pathways regulate the cell’s cytoskeleton and calcium stores [17]. All Wnt signaling pathways are initiated by the binding of a Wnt ligand to a Frizzled family transmembrane receptor. In the case of the canonical pathway, the resulting intracellular signaling cascade leads to the inactivation of a β-catenin destruction complex [18]. β-catenin thus avoids destruction and translocates from the cytoplasm to the nucleus where it interacts with TCF7L2 and other transcription factors, replacing transcriptional repressors and recruiting coactivators [17,19].

Genes involved in the β-catenin destruction complex may influence susceptibility to type 2 diabetes, given the critical role this complex plays in Wnt signal transduction with the resulting downstream effects on diabetes locus *TCF7L2*. AA as well as European individuals may be affected, considering that the *TCF7L2* / diabetes association is seen across racial groups. Under one scenario, gene mutations might render the β-catenin destruction complex inactive at all times. In this situation, β-catenin would avoid destruction even in the absence of Wnt ligands, thereby accumulating in the cytoplasm and nucleus and binding to TCF7L2 and other transcription factors. These transcription factors would then act mostly as transcriptional activators, and overexpression of some of their target genes may lead to diabetes pathology. With this type of scenario in mind, the present study was initiated to investigate genes involved in the β-catenin destruction complex for evidence of variants that may impact risk of type 2 diabetes in AA women. Given the small effect sizes generally seen for common susceptibility variants, the present analyses utilized gene-based testing in an attempt to identify important genes with multiple risk variants that might otherwise be missed in a SNP-based approach.

## Methods

### Study population

The data source for the current analyses was the Black Women’s Health Study (BWHS) [20], a prospective cohort study of health and illness among U.S. black women that began in 1995 when 59,000 African American women 21–69 years of age from across the U.S. completed a 14-page postal health questionnaire. Biennial follow-up questionnaires ascertain new cases of type 2 diabetes and other health outcomes and update covariate data. Through 2013, follow-up had been completed for 88% of the potential years of follow-up for the baseline cohort. The BWHS was granted approval by the Institutional Review Board of Boston University, and all study subjects provided written informed consent.

The accuracy of self-reported diabetes in the BWHS was previously assessed using medical records from a sample of 227 women who reported this diagnosis [21]. Type 2 diabetes was confirmed in 96% of these women, and another 2% were found to have other types of diabetes. The prevalence of undiagnosed diabetes in the BWHS was also previously assessed [22], using data from collected blood samples. Of the 1873 cohort members who provided a blood sample in the first year of blood collection and had never reported diabetes, 120 (6.4%) had HbA_1c_ levels of 6.5% (47.5 mmol/mol) or higher, meeting criteria for diabetes [23].

About 50% of BWHS study participants provided DNA samples for analysis, and these subjects were found to be highly representative of all BWHS participants across a number of factors including geographic region, education, and BMI. A case-control sample was drawn from among participants with DNA samples for genotyping and analysis: incident cases of type 2 diabetes were selected, and one control was matched to each case on birth year (+/- 2 years) and geographic region of residence.

We sought replication of the top associations from the BWHS in up to 8284 African American cases and 15,543 controls from the MEDIA (Meta-analysis of type 2 diabetes in African Americans) Consortium, which has been previously described [9]. MEDIA includes 17 African American type 2 diabetes GWAS.

### SNP selection

The Reactome database [24,25] (http://www.reactome.org/) was used to identify 68 genes that code for proteins involved in the Wnt pathway’s β-catenin destruction complex. Tag SNPs were then selected for each of these 68 genes (+/- 20 kb regions surrounding them) in order to capture (at r^2^ ≥ 0.9) all SNPs with minor allele frequency (MAF) ≥ 5%, based on the African populations in 1000 Genomes [26] (http://www.1000genomes.org/). In addition, tag SNPs were selected for the +/- 100 kb region surrounding the *TCF7L2* index SNP rs7903146.

### Genotyping and QC

Genotyping of the selected Wnt pathway SNPs was performed in two batches totaling 6080 samples (including duplicates), as part of a custom Affymetrix Axiom array that contained 45,747 SNPs chosen for several type 2 diabetes projects. The Axiom array data underwent extensive QC procedures carried out by Affymetrix and Slone Epidemiology Center. About 13% of samples were removed due to high missing call rates (defined as >5%), poor reproducibility, or Dish-QC values <0.6. About 17% of SNPs were removed due to poor cluster properties, high missing call rates (defined as >10%), deviation from Hardy-Weinberg equilibrium (p <10^−5^ in controls), or high rates of discordant calls across duplicate samples. Only SNPs that passed QC in both sample batches were retained for analyses. After the application of these QC filters and the consolidation of 63 expected and confirmed duplicate sample pairs, the full type 2 diabetes data set contained 5228 subjects (2632 cases and 2596 controls) and 38,008 SNPs, including 3430 SNPs selected for the current analyses of Wnt pathway genes.

### Imputation

After prephasing the study data with SHAPEIT version 2 [27], imputation was performed using the IMPUTE2 software [28] and the 1000 Genomes Phase 3 data as the reference panel (5/2/2013 1000 Genomes data, October 2014 haplotype release). Imputation resulted in a total of 32,165 Wnt pathway SNPs with MAF ≥ 0.5% and imputation info score ≥ 0.5 for analysis. The imputation info score used for SNP filtering was the imputation metric produced by IMPUTE2 [29].

### Association analysis

We first computed genotype principal components using the smartpca program in the EIGENSOFT package [30], based on 18,825 genotyped and pruned common (MAF >5%) SNPs in the full type 2 diabetes data set. The principal components of genotype were tested for association with case status after accounting for the study covariates: age at baseline, geographical region, and genotyping batch. For all association analyses, we included principal components that had p <0.1 in this multivariable model.

Gene-based association analyses were conducted using the adaptive rank truncated product (ARTP) statistic [31], as implemented in the R package ARTP2 [32]. The ARTP method was selected for its ability to optimize the number of single SNP p-values combined in each gene-based test. According to the options we set, the ARTP2 program selected an optimal test for each gene using between one and 10 SNPs per gene. All genotyped and imputed Wnt pathway SNPs were input into ARTP2 for analysis. Based on the program parameters chosen, ARTP2 removed 10,445 SNPs with MAF <2% in order to eliminate low frequency, imputed SNPs. Next, it identified pairs of SNPs with linkage disequilibrium (LD) r^2^ >0.8 within each gene and removed the SNP with the lower MAF from each pair, resulting in removal of 14,918 SNPs. After implementation of the MAF and LD filters, 6802 SNPs remained for gene-based analysis.

Single SNP association tests, required as input for gene-based testing, were performed using logistic regression analyses of the imputed dosage genotype data. All statistical models were adjusted for age at baseline, geographical region, genotyping batch, and genotype principal components.

## Results

The results of the gene-based analyses are shown in Table 1. One gene, *PSMD2*, was significantly associated with the risk of type 2 diabetes after a Bonferroni correction for the 69 genes tested (nominal p = 2.2 x 10^−4^, corrected p = 0.016). One other gene, GWAS locus *TCF7L2*, was associated with a nominal p <0.01 (p = 1.5 x 10^−3^), but this result did not survive a correction for multiple testing.

**Table 1:** Associations of Wnt pathway genes with risk of type 2 diabetes in the BWHS.

Gene	Chromosome	Number of SNPs in the analysis	P-value
*PSMD2*^a^	3	51	2.2 x 10^−4^
*TCF7L2*	10	368	1.5 x 10^−3^
*PSMD11*	17	53	0.017
*CSNK1A1*	5	104	0.025
*FRAT1*	10	62	0.033
*PSME2*	14	68	0.042
*PSME1*	14	59	0.051
*PSMD4*	1	40	0.095
*PSMD14*	2	49	0.11
*PSMB1*	6	67	0.12
*PSMD12*	17	86	0.15
*PPP2R5D*	6	85	0.15
*PSMD10*	23	11	0.15
*PSME4*	2	148	0.15
*UBC*	12	154	0.17
*PSMC1*	14	97	0.17
*UBA52*	19	96	0.18
*FRAT2*	10	62	0.19
*PSMB10*	16	33	0.19
*CTNNB1*	3	123	0.22
*PPP2R1B*	11	74	0.23
*APC*	5	183	0.23
*PPP2R5C*	14	375	0.26
*PSMB6*	17	105	0.27
*PSMB3*	17	128	0.28
*PSMA2*	7	53	0.30
*PPP2R5E*	14	381	0.30
*PSMD3*	17	121	0.31
*PPP2R1A*	19	144	0.32
*PSMC2*	7	65	0.33
*PSMB8*	6	135	0.35
*PSMB9*	6	141	0.36
*GSK3B*	3	182	0.37
*BTRC*	10	110	0.37
*UBB*	17	64	0.39
*PSMD6*	3	103	0.40
*PSMA6*	14	88	0.40
*PSMC5*	17	29	0.40
*PSMD5*	9	51	0.40
*AMER1*	23	14	0.40
*PSMA8*	18	90	0.40
*PSMA7*	20	65	0.41
*PSMD13*	11	189	0.42
*PSMD1*	2	121	0.42
*PSMC3*	11	58	0.51
*PPP2CB*	8	109	0.55
*RPS27A*	2	35	0.57
*PSMA3*	14	48	0.60
*PSMB4*	1	51	0.61
*PSMA5*	1	31	0.64
*PSMC4*	19	42	0.67
*PSMD8*	19	62	0.68
*PSMC6*	14	77	0.75
*CUL1*	7	218	0.81
*PSMA1*	11	95	0.83
*PSMD7*	16	46	0.83
*PSMA4*	15	61	0.83
*PSMF1*	20	194	0.84
*PSMB2*	1	77	0.86
*PPP2R5A*	1	81	0.87
*AXIN1*	16	378	0.89
*PPP2R5B*	11	59	0.90
*PPP2CA*	5	60	0.91
*PSMB5*	14	120	0.96
*PSME3*	17	40	0.96
*PSMB11*	14	107	0.96
*SKP1*	5	83	0.96
*PSMD9*	12	61	0.97
*PSMB7*	9	134	0.99

^a^ Gene *PSMD2* remains significant after a Bonferroni correction for the 69 genes tested (corrected p = 0.016).

Table 2 shows the genetic variants that were included in the best models selected for genes *PSMD2* and *TCF7L2*. The best model selected for the *PSMD2* region included nine genetic variants. The best model selected for *TCF7L2* included three genetic variants. The most significantly associated variant in the *TCF7L2* region was the GWAS index SNP rs7903146 (p = 1.0 x 10^−5^), which was associated with a ~20% increased risk of type 2 diabetes (OR 1.21, 95% CI 1.11, 1.32).

**Table 2:** Genetic variants comprising the optimal models for *PSMD2* and *TCF7L2*: associations with risk of type 2 diabetes in the BWHS.

Variant	Gene^a^	Reference allele	Minor allele	Minor allele frequency (%):			Imputation r2^b^	OR (95% CI)	P-value
				BWHS	AFR	EUR			
*PSMD2* region									
rs55808452	*ECE2*	GGCAAAGGGTGG	-	13.0	NA	NA	0.70	0.75 (0.65, 0.87)	1.0 x 10^−4^
rs7635741	*EIF4G1*	G	T	8.1	3.5	22.8	0.61	0.69 (0.56, 0.84)	2.2 x 10^−4^
rs1879244	*EIF4G1*	T	C	7.1	1.7	24.7	0.95	0.72 (0.60, 0.86)	2.4 x 10^−4^
rs939317	*EIF4G1*	G	A	9.0	4.1	24.7	genotyped	0.76 (0.65, 0.88)	2.6 x 10^−4^
rs9846954	*EIF4G1*	T	A	48.8	42.0	75.1	genotyped	0.87 (0.80, 0.94)	4.2 x 10^−4^
rs2376524	*EIF2B5*	A	C	7.6	2.7	23.2	0.93	0.75 (0.63, 0.88)	5.8 x 10^−4^
rs9883929	*PSMD2*	A	C	18.2	22.4	8.0	0.91	1.19 (1.07, 1.32)	9.6 x 10^−4^
rs1687230	*ECE2*	T	C	16.5	11.1	37.0	genotyped	0.84 (0.75, 0.94)	2.2 x 10^−3^
rs72591978	*EIF2B5*	CA	C	46.5	NA	NA	1.01	0.89 (0.82, 0.96)	2.9 x 10^−3^
*TCF7L2* region									
rs7903146	*TCF7L2*	C	T	28.2	26.0	31.7	genotyped	1.21 (1.11, 1.32)	1.0 x 10^−5^
rs34872471	*TCF7L2*	T	C	33.6	31.2	31.5	1.02	1.20 (1.10, 1.30)	1.6 x 10^−5^
rs114770437	*TCF7L2*	G	A	7.8	9.1	0.0	genotyped	0.73 (0.62, 0.85)	7.7 x 10^−5^

Abbreviations: BWHS, Black Women’s Health Study controls; AFR, 1000 Genomes Project Phase 3 African samples; EUR, 1000 Genomes Project Phase 3 European samples; OR, odds ratio (adjusted for age at baseline, geographical region, genotyping batch, and genotype principal components); CI, confidence interval; NA, not applicable (allele frequencies are not available in 1000 Genomes).

^a^ All SNPs are intronic.

^b^ The imputation r^2^ quality metric computed by PLINK version 1.90 [33] (https://www.cog-genomics.org/plink2).

Although we had removed correlated SNPs prior to gene-based testing, the r^2^ threshold used was 0.8, and there was moderate LD (0.45 < r^2^ < 0.8) in the study sample among five of the top six variants in the best model for *PSMD2*. Nevertheless, the nine variants included in the best model for *PSMD2* comprised four distinct LD groups, using r^2^ = 0.35 as the cutoff for LD grouping. For *TCF7L2*, there was moderate LD between the top two variants in the best model (rs7903146 and rs34872471 had r^2^ = 0.77), but rs114770437 was not correlated (r^2^ <0.05 with each of the top two SNPs).

We next reviewed SNPs in the *PSMD2* and *TCF7L2* regions that had been removed by ARTP2 during pruning, in case any of the excluded SNPs were of interest due to potential functionality. In the +/- 20 kb region surrounding *PSMD2*, a genotyped missense SNP rs2178403 (A/G, Met/Val), located in gene *EIF4G1* and excluded from gene-based analyses due to its high LD (r^2^ = 0.93) with SNP rs1879244 (Table 2), was associated with diabetes risk, with a p-value smaller than that of the nine SNPs from the best model (p = 8.0 x 10^−5^). The A allele of rs2178403 had a frequency of 6.6% in the study controls and was associated with a 30% decreased risk of type 2 diabetes (OR 0.70, 95% CI 0.58, 0.83).

Given that the most significant SNP in the *PSMD2* region was potentially functional and the top SNP in *TCF7L2* was the GWAS index SNP, we assessed how much of the association signal in each region was driven by these top SNPs. We reran single variant analyses in these two regions, conditioning on those SNPs. The results of the conditional analyses are shown in Table 3. When we conditioned on rs2178403, three of the nine variants in the best model for *PSMD2* remained nominally significant (p <0.05). When we conditioned on rs7903146, SNP rs114770437 in *TCF7L2* remained nominally significant (p = 1.3 x 10^−3^). Thus, both regions may contain multiple independent signals.

**Table 3:** Genetic variants comprising the optimal models for *PSMD2* and *TCF7L2*: analyses conditioning on the top SNP in each region.

Variant	Reference allele	Minor allele	OR	P-value	Conditional OR^a^^,^^b^	Conditional p-value	Comment
*PSMD2* region							
rs2178403	G	A	0.70	8.0 x 10^−5^	NA	NA	excluded from gene-based analysis during pruning
rs55808452	GGCAAAGGGTGG	-	0.75	1.0 x 10^−4^	0.85	0.14	
rs7635741	G	T	0.69	2.2 x 10^−4^	0.92	0.71	
rs1879244	T	C	0.72	2.4 x 10^−4^	1.19	0.60	
rs939317	G	A	0.76	2.6 x 10^−4^	0.90	0.40	
rs9846954	T	A	0.87	4.2 x 10^−4^	0.90	8.6 x 10^−3^	
rs2376524	A	C	0.75	5.8 x 10^−4^	1.00	0.98	
rs9883929	A	C	1.19	9.6 x 10^−4^	1.17	3.3 x 10^−3^	
rs1687230	T	C	0.84	2.2 x 10^−3^	0.91	0.16	
rs72591978	CA	C	0.89	2.9 x 10^−3^	0.92	0.039	
*TCF7L2* region							
rs7903146	C	T	1.21	1.0 x 10^−5^	NA	NA	
rs34872471	T	C	1.20	1.6 x 10^−5^	1.08	0.36	
rs114770437	G	A	0.73	7.7 x 10^−5^	0.77	1.3 x 10^−3^	

Abbreviations: OR, odds ratio (adjusted for age at baseline, geographical region, genotyping batch, and genotype principal components); NA, not applicable.

^a^ Conditional odds ratios for variants in the *PSMD2* region are adjusted for age at baseline, geographical region, genotyping batch, genotype principal components, and SNP rs2178403.

^b^ Conditional odds ratios for variants in the *TCF7L2* region are adjusted for age at baseline, geographical region, genotyping batch, genotype principal components, and SNP rs7903146.

A haplotype analysis of rs7903146 and rs114770437 in *TCF7L2* showed the presence of only three of the four possible haplotypes including common haplotype rs7903146-C / rs114770437-G (63%), and haplotypes T/G (30%) and C/A (7%). An omnibus test assessing the joint effect of all haplotypes on the risk of type 2 diabetes was significant with p = 2.5 x 10^−7^. Compared to the C/G haplotype, the T/G haplotype was associated with an 18% increased risk of type 2 diabetes (OR 1.18, 95% CI 1.09, 1.29), and the C/A haplotype was associated with a 23% reduction in risk (OR 0.77, 95% CI 0.66, 0.90) (Table 4).

**Table 4:** Haplotype analysis of SNPs rs7903146 and rs114770437 in *TCF7L2*.

rs7903146 allele	rs114770437 allele	Haplotype frequency (%)		OR (95% CI)	P-value
		Cases (n = 2632)	Controls (n = 2596)		
C	G	62.3	64.1	1.00 (reference)	reference
T	G	31.7	28.1	1.18 (1.09, 1.29)	5.7 x 10^−5^
C	A	6.0	7.8	0.77 (0.66, 0.90)	1.3 x 10^−3^

Abbreviations: OR, odds ratio (adjusted for age at baseline, geographical region, genotyping batch, and genotype principal components); CI, confidence interval.

We sought replication in the MEDIA Consortium for the top SNPs in the *PSMD2* region and for the potentially novel risk variant rs114770437 in *TCF7L2*. Replication data were not available for rs114770437, but data were available for four of the nine variants in the best model for *PSMD2* (rs939317, rs9846954, rs2376524, and rs1687230). In MEDIA, these four variants had effect estimates pointing in the same direction as BWHS, but the odds ratios were quite small (≤ 1.08 for the risk alleles), and none of the associations were statistically significant (p >0.05). Results of a meta-analysis combining BWHS and MEDIA for these four SNPs are shown in S1 Table.

## Discussion

Gene-based analyses of common variants in the vicinity of β-catenin destruction complex genes identified an association between the *PSMD2* gene region and type 2 diabetes in 2632 AA cases and 2596 AA controls. Eight of the nine variants in the best model for *PSMD2* were not located within *PSMD2* itself but were instead located within other surrounding genes on chromosome 3q27.l (*EIF4G1*, *ECE2*, and *EIF2B5*) (Table 2). The most significant variant in the *PSMD2* region, missense SNP rs2178403, is located within a plausible diabetes candidate gene, *EIF4G1*. *EIF4G1* encodes a component of the multi-subunit protein complex EIF4F. The EIF4F complex facilitates recruitment of mRNA to the ribosome, which is the rate-limiting step in protein synthesis. There is evidence that compromised insulin signaling in pancreatic beta cells downregulates *EIF4G1*, leading to the inhibition of carboxypeptidase E (CPE) expression, with a subsequent reduction of proinsulin processing and a corresponding increase in the levels of circulating proinsulin [34].

While *EIF4G1* is a potential susceptibility gene, the results of our analyses conditioning on rs2178403 suggest that the association signal in the *PSMD2* region, if valid, may not be fully captured by variants in *EIF4G1* alone. Furthermore, other genes in this region, including *PSMD2* itself, could be linked to diabetes pathology. The *PSMD2* gene was included in this study because of its involvement in the Wnt pathway’s β-catenin destruction complex: *PSMD2* encodes a regulatory subunit of the 26S proteasome, and it is the 26S proteasome that carries out the actual destruction of β-catenin (as well as other ubiquitinated proteins) [35,36]. It has been shown that a high fat diet downregulates hepatic transcription of *PSMD2* in mice that are resistant to the development of insulin resistance and non-alcoholic fatty liver disease (NAFLD), while upregulating transcription in mice with susceptibility to developing insulin resistance and NAFLD [37].

*ECE2* is another possible susceptibility gene near *PSMD2*. The enzyme encoded by *ECE2* converts big endothelin-1 to the vasoconstrictor endothelin-1, and is involved in the processing of several neuroendocrine peptides. This enzyme may also act as a methyltransferase. A mouse study reported an association between hyperglycemia at an early stage of autoimmune diabetes and downregulation of *ECE2* transcription in the kidneys [38]. In our study, the most significant variant in the top model for the *PSMD2* region, rs55808452, was located within an intron of *ECE2*, although it should be noted that this variant was in moderate LD with several *EIF4G1* variants including missense SNP rs2178403 (r^2^ = 0.54).

It should be acknowledged that the association we observed for the *PSMD2* region may very well be a false positive result given that four of the top variants in this region failed to replicate in the large AA sample from the MEDIA Consortium. In addition, four of the five variants that were not available in MEDIA were in moderate LD (0.7 < r^2^ < 0.8) with at least one of the SNPs that failed replication. If SNPs in this region are truly associated, they likely have small effects as represented by the MEDIA estimates. Although the MEDIA estimates were close to the null (odds ratios between 0.92 and 0.98), they were all in the same direction as our study. Thus, the possibility of true, small effects does exist.

Apart from the *PSMD2* region, the other interesting finding from the present study concerned the GWAS gene *TCF7L2*. SNP rs114770437 (BWHS MAF = 7.8%) was one of three variants included in the best gene-based model for *TCF7L2* and was not correlated with the GWAS index SNP rs7903146 (BWHS MAF = 28.2%). The minor A allele at rs114770437 was associated with a 27% reduction in the risk of type 2 diabetes. The association with this SNP remained nominally significant after control for rs7903146 (conditional OR = 0.77; p = 1.3 x 10^−3^). Thus, rs114770437 may represent an independent association signal in *TCF7L2* in AA populations. SNP rs114770437 is monomorphic in 1000 Genomes European samples, and this may explain the results of a Bayesian fine mapping analysis by the Wellcome Trust Case Control Consortium (WTCCC), which suggested that no such secondary signal exists in *TCF7L2* in Europeans [39]. In the WTCCC study, the posterior probability that rs7903146 was driving the *TCF7L2* association signal was 75%. An additional 13% of the posterior probability was accounted for by correlated SNP rs34872471, the second most significant SNP in the best model for *TCF7L2* in our study. No other SNP accounted for more than 3% of the posterior probability.

Despite a respectable sample size of 2632 AA cases and 2596 AA controls, the present study had limited power to detect individual SNP associations. Still, we replicated the association of the *TCF7L2* GWAS index SNP rs7903146. The failure of *TCF7L2* to achieve significance in our gene-based analyses is likely due to the inherent power limitations of the ARTP gene-based approach in situations where much of a gene’s association is driven by a single SNP. In our application of the ARTP method, each gene test had to correct for having considered up to 10 SNPs. Another limitation of our study was the use of imputed genotypes for many SNPs. However, SNPs with an imputation info score <0.5 or MAF <2% were excluded from the association analyses in order to improve the accuracy of the data used. Lastly, non-differential misclassification of diabetes in our sample, though likely to be small, may have resulted in underestimation of the associations.

In summary, we observed a significant association between the *PSMD2* gene region and type 2 diabetes in women of African ancestry in a gene-based analysis. This finding opens the possibility that *PSMD2*, a gene involved in the Wnt pathway’s β-catenin destruction complex, or another nearby gene such as *EIF4G1* or *ECE2*, may be a susceptibility locus for type 2 diabetes. It is also possible that the observed association is a false positive result, given the failed replication of a subset of the top SNPs in this region. Our analyses also suggested a possible association signal in *TCF7L2* that is independent of the GWAS index SNP rs7903146 and may be present only in AA populations. Replication is needed in additional AA samples in order to validate our findings.

## Supporting information

S1 Table Meta-analysis of BWHS and MEDIA for the *PSMD2* region. (DOCX)
