# Supplementary material for: A novel TCF7L2 type 2 diabetes SNP identified from fine mapping in African American women
Source: PLoS One. 2017 Mar 2;12(3):e0172577. doi: 10.1371/journal.pone.0172577 (PMC5333820; doi:10.1371/journal.pone.0172577)
Supplement: S1 Table — (DOCX) [file pone.0172577.s001.docx]

| **S1 Table. Meta-analysis of BWHS and MEDIA for the *PSMD2* region** | | | | | | |
| --- | --- | --- | --- | --- | --- | --- |
| SNP | BWHS | | MEDIA | | BWHS + MEDIA | |
|  | OR (95% CI) | P-value | OR (95% CI) | P-value | OR (95% CI) | P-value |
| rs939317 | 0.76 (0.65, 0.88) | 2.6 x 10^-4^ | 0.94 (0.84, 1.04) | 0.23 | 0.87 (0.80, 0.95) | 1.9 x 10^-3^ |
| rs9846954 | 0.87 (0.80, 0.94) | 4.2 x 10^-4^ | 0.97 (0.92, 1.02) | 0.26 | 0.94 (0.90, 0.98) | 4.1 x 10^-3^ |
| rs2376524 | 0.75 (0.63, 0.88) | 5.8 x 10^-4^ | 0.92 (0.82, 1.04) | 0.18 | 0.86 (0.78, 0.95) | 2.1 x 10^-3^ |
| rs1687230 | 0.84 (0.75, 0.94) | 2.2 x 10^-3^ | 0.98 (0.92, 1.04) | 0.48 | 0.94 (0.89, 1.00) | 0.033 |
|  | | | | | | |
